# Is the solution composition and external magnetic field influencing FeNi film deposition – physicochemical and tribological studies

**DOI:** 10.1038/s41598-024-84014-x

**Published:** 2025-04-03

**Authors:** Anna Maria Białostocka, Marcin Klekotka, Urszula Klekotka, Beata Kalska-Szostko

**Affiliations:** 1 Faculty of Electrical Engineering, BialystokUniversity of Technology, Wiejska 45D, 15-351 Białystok, Poland; 2 Faculty of Mechanical Engineering, Bialystok University of Technology, Wiejska 45C, 15-351 Białystok, Poland; 3 Faculty of Chemistry, University of Bialystok, Ciołkowskiego 1K, 15-245 Białystok, Poland

**Keywords:** Electrocrystallization, External magnetic field, Magnetohydrodynamics, Substrate, Thin films, Tribology, Engineering, Materials science, Nanoscience and technology, Physics

## Abstract

The iron-nickel films were deposited on the two metallic substrates (Cu, CuZn) from a mixture of iron and nickel salts and in the external magnetic field (EMF) presence. The films were electrodeposited in the fixed value of an electric current density and the time conditions. The EMF orientation and the electrochemical bath composition—molar ratio of Fe:Ni were changeable, parallel or perpendicular, and 1:1 or 1:2, respectively. Examined layers present variable surface morphologies and compositions depending on the substrates and the presence or absence of the EMF. Layers quality was analyzed using: scanning electron microscopy, energy dispersive X-ray spectroscopy, and X-ray diffraction. Tribo-mechanical properties (roughness, wear tracks) were registered using a confocal laser scanning microscope. The observed results show that a variation in the surface morphology and the alloy composition is related to the electrolyte composition (Fe:Ni ratio) and the presence or absence of EMF. Higher Fe content in the electrolyte (Fe:Ni, 1:1) causes more significant anomalous deposition and an increase of tribo-mechanical parameters. The tribological parameters (S_sk_, S_ku_, COF) depend on the electrolyte composition and substrate’s properties as a result of the film’s growth condition. The reduction in the thickness of the deposited layer (both electrolyte’s ratios) after applying an external magnetic field is the result of the increase of roughness parameters.

## Introduction

In recent years, thin films have attracted much attention among scientists and industries. Especially interesting become FeNi coatings due to their unique magnetic, mechanical, chemical, and physical properties. Besides that, applying an external magnetic field during the electrodeposition process has been the subject of many studies worldwide. Their interdisciplinary potential results in the development of materials with controlled morphologies (grain size, thickness), roughness, crystal structures, and compositions, which could be applied in many branches of industry. Electrodeposition is a widely used method in the production of metallic coating alloys due to low process costs and neutral conditions. A better understanding of this flexible method is a milestone towards the development of a new material known as „tailor-made”. Their applications range from the production of energy through military and space research to biomedicine and biotechnology, which are very important from the modern world point of view. The alloy’s properties are affected by processing variables such as electrolyte composition, temperature, current density, mixing conditions, substrate surface quality, etc.^1–4^. In many research, besides main electrolyte components, more and more attention is paid to particular additives that significantly impact both the parameters of the process itself (e.g., current) and the properties of the layers obtained (e.g., crystal structure, composition, adhesion). Boric acid, ascorbic acid, or SDS can be found among such components. Their effect has been widely analyzed in many papers^5^. The crystal nucleation and the layer’s growth (especially the early stages) are particularly important for thin film deposition and technology development^6^. Applying the external magnetic field (EMF) during electrodeposition leads to a macroscopic stirring of the electrolyte (called as micro-magnetohydrodynamic effect)^7^. The additional convection (reduction in the diffusion layer thickness at the electrode surface) results in a limited current density increase and also changes the nucleation’s rate. In the situation when B (magnetic field) is parallel to E (electric field), the microturbulence suppress the nucleation, and a specific morphology is generated^8,9^. The microturbulences will appear due to local Lorentz forces (${F}_{L}$) acting around the nucleation center. When B is perpendicular to E, ${F}_{L}$ is maximal^10–15^. The effects of interaction or counteraction of the fields are visible in the form of fine-grained or coarse-grained structures and, therefore, a surface with higher or lower roughness. The EMF affects both parts of the electrodeposition process, which is the growth and nucleation of the obtained alloys. Some investigators have found that the nucleation process perfectly describes two parameters: *A*—nucleation rate constant and *N*_0_—nucleation site density. Instantaneous nucleation (*A* ≫ 1) is characterized by the high growth rate of a new phase and a small number of formed active nucleation sites. Progressive nucleation (*A* ≪ 1) implies a slow growth rate and a large number of new nuclei formed, respectively. An increase in nucleation site density results in a decrease in the growth rate^16^. The nucleation depends on the metal’s interaction with the substrate, which influences the next steps of the layer’s growth. The first affects the adhesive strength in the interface of film/substrate, which is correlated with the crystallographic coherency—eutectic (poor adhesion) or peritectic (favorable adhesive strength) alloy system^17^. The study of the proper adhesion problem has attracted much attention due to its practical application in the electronic industry, epsecially^12,17^. The tribological properties like friction (depth and volume of friction marks, coefficient of friction), wear (wear tracks), microhardness, or roughness (skewness, kurtosis) are of great importance due to their influence on the increase the efficiency of mechanical systems. Electrodeposition is a well-established technique that produces dense, uniform, adherent metals, alloys, and composite materials. The electric current forces the solvated species to be transported from the solution to the cathode surface, where they are incorporated and reduced in the form of 2D or 3D deposits. Their densities influence the mechanism of layer growth. The instantaneous results in rougher surface morphology but a progressive flatter one. In the case of the FeNi alloy deposition, an „anomalous co-deposition”, where the less noble metal’s percentage in the deposit is higher than its presence in the electrolyte, can be observed^18^. The electroplating conditions themselves are critical, including physicochemical and electrical parameters for particular layer quality deposition. Therefore, their proper selection influences the morphology, structure, and composition of the obtained alloy and the nature of the process itself (anomalous or normal co-deposition). The existence of an anomalous electroplating type (Matlosz’s definition) is a consequence of the Fe:Ni ratio of electrolyte (its pH also)^19^. According to Su and Qiang, regardless of the Ni^2+^: Fe^2+^ molar ratio, the Ni content in the deposited alloys was always lower than that in the electrolyte. This was caused by preferential absorption of iron hydroxide on the cathode (high concentration of FeOH^+^ near the electrode). The radius of the Ni atom (0.162 nm) is bigger than the radius of Fe (0.110 nm) and the increase of the nickel content in the FeNi alloy results in bigger lattice parameter, what was confirmed by XRD^18^. The consequence of the larger grains in the films is changes in pH values^20–22^. Another study (Moniruzzaman et al.) states that after increasing the Ni:Fe ratio in the bath, the occurred layers have increased Ni content and also increased magnetic properties (saturation magnetization—M_s_). However, it is depend on the complexing agents application and Brenner’s definition of anomalous codeposition. The authors conclude that this kind of deposition takes place, and only the addition of the complexing agent suppresses it^23^. The higher Ni:Fe ratio yields a higher hardness of the film, which depends directly on the current density. The complexing agent in the bath relieves the stress, and the surface is fine-grained (smooth). The morphology of the surface can be controlled by electrolyte composition—the presence of the complexing agents with proper ratio^23^. In the work of Abdel-Karim et al., the researchers found a relationship between the Ni:Fe ratio in the solution and the morphology of the resulting alloy (iron content increasing led to well-defined, finer nodular particles formation)^1^. In other reference, Su and Qiang, the size of the crystals depended on the amount of nickel in the alloy^20^. They also reached similar conclusions to Moniruzzaman et al. regarding the microhardness of sediments^12^. A higher Ni:Fe molar ratio film exhibits better electrocatalytic activity, which the authors explain by the ultra fine microstructure (the low crystallinity structure) and the increased density of active surface sites^24–33^. Surface energy decides the film growth mechanism, which was confirmed in many research works (island or layer-by-layer mechanism). What reflects in tribological properties (friction, wear, lubrication, etc.) and also should not be neglected—R_sk_ (sensitive on the deep valleys), R_ku_ (informs on the sharpness density of the profile), R_vk_ (valley depth) and R_pk_ (peak height)^34–36^. The increase of the surface roughness of the coatings results in cracks, chipping, and pitting. The roughness influences the friction and the wear rate more significantly than the coating materials^36,37^. Therefore, the influence of the electrolyte composition and the external magnetic field presence is still a hot subject of interest for many scientists worldwide. The continuous expansion of knowledge in this area results in a deeper knowledge of the details of the deposition process itself and surface finishing, which is so important from the industrial and economic point of view.

## Materials and methods

**Fig. 1:**
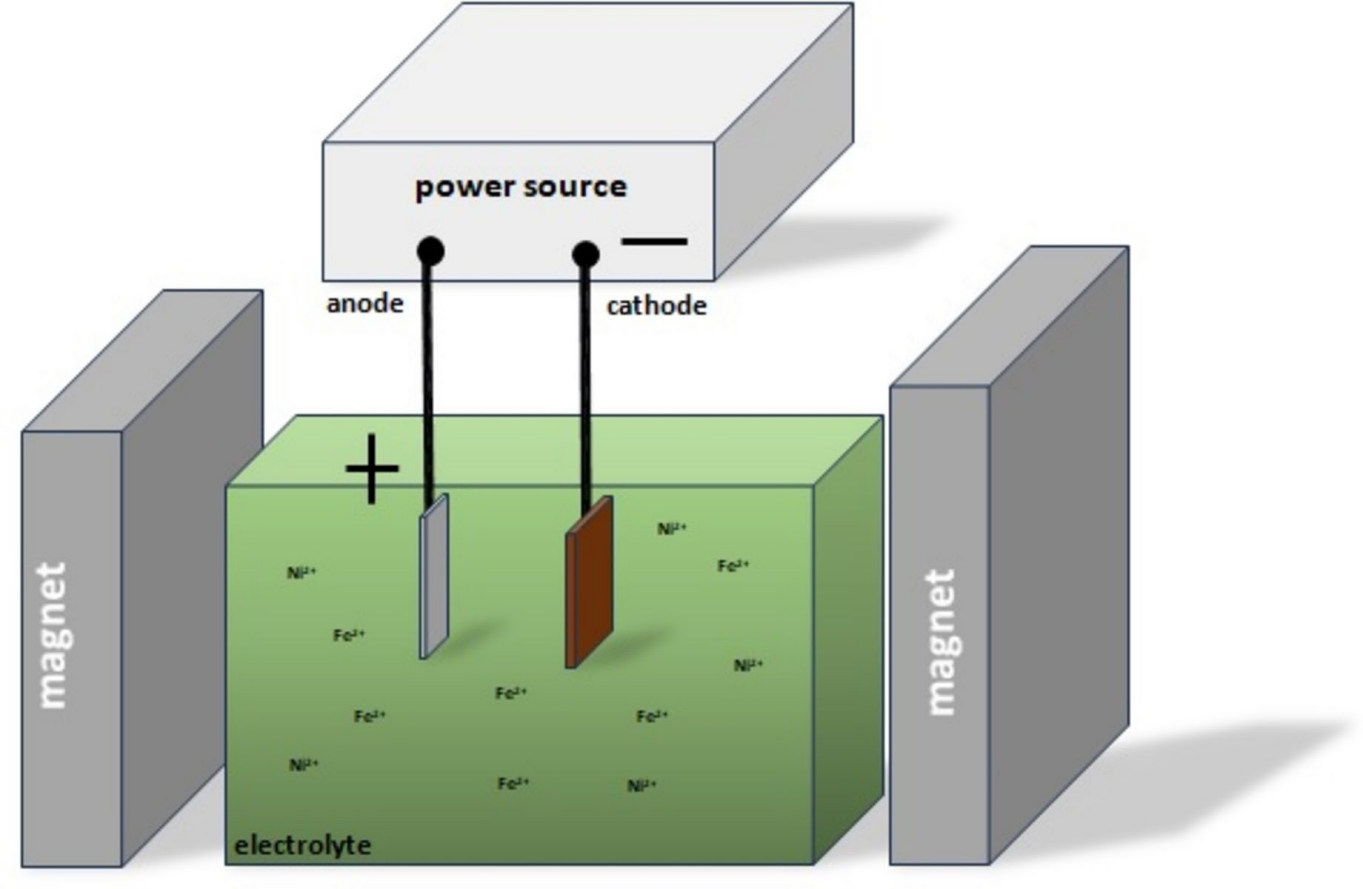
Schematic presentation of the deposition set-upside view (parallel arrangement of the magnetic and electric field).

The two combinations of Fe:Ni molar ratio in electrolyte, 1:1 (first composition) and 1:2 (second composition), and two commercially available substrates (Cu and CuZn) were used for the thin film deposition^17^. The experimental deposition bath consisted of FeSO_4_·7H_2_O, NiSO_4_·7H_2_O and H_3_BO_3_. Each experiment was performed for a determined time of 1 h (3600 s) in the presence or absence of the external magnetic field (EMF). A set of two factory-made NdFeB permanent magnet plates (IBS Magnet, 37.5 cm^3^ volume) of about 1 T were fixed at a distance of 55 mm. The strength of the magnets was measured (gauge FH51, Magnet-Physik) and occurred to be in the range of 80 mT to 200 mT in the volume of electrodeposition. The electrodeposition set-up consisted of a beaker, a D.C. power supply (Matrix MPS 7163), 20 ml of electrolyte, and electrodes. The platinum anode (0.3 cm^2^ of area) and cathode (1.5 cm^2^ of area) were placed parallel, and the distance between them was kept constant—20 mm. All of the experiments were performed at room temperature (21 °C) under galvanostatic conditions by applying current density at 50 mA/cm^22^. The laboratory set up is depicted in Fig. 1. The surface morphology and the thickness of the films were examined with a Scanning Electron Microscope (SEM) (INSPEC S60, FEI). An energy-dispersive X-ray spectrometer incorporated with SEM gave the knowledge about the film composition. X-ray diffraction (XRD) (Agilent Technologies SuperNova diffractometer with a micro-focused Mo source, Kα2 = 0.0713067 nm) was carried out by diffractometer equipped with a microfocus Mo Kα radiation source (λ = 0.713067 Å). It provided information about the crystal structure of the FeNi alloys. The tribological measurements were made using a UMT TriboLabtribometer with a ball-on-disc system under dry friction conditions, and each of them took 300 s. The reciprocating motion of a 6 mm corundum ball with an amplitude of 500 μm was repeated 3 times, with the following parameters: F_n_ = 2 N, f = 10 Hz. The use of a confocal laser scanning microscope (LEXT OLS 4000) results in the analysis of the thickness, volume, and depth of wear tracks. The optical method was used to measure the roughness of the probes. The values of parameters S_sk_ (skewness) and S_ku_ (kurtosis) were calculated to achieve a useful understanding of the surface’s tribological development. Microhardness occurred using the Vickers method—a load of 0.9807N and repeated 10 times^21,38^.

## Results and discussion

### Morphology determination—scanning electron microscopy

**Fig. 2:**
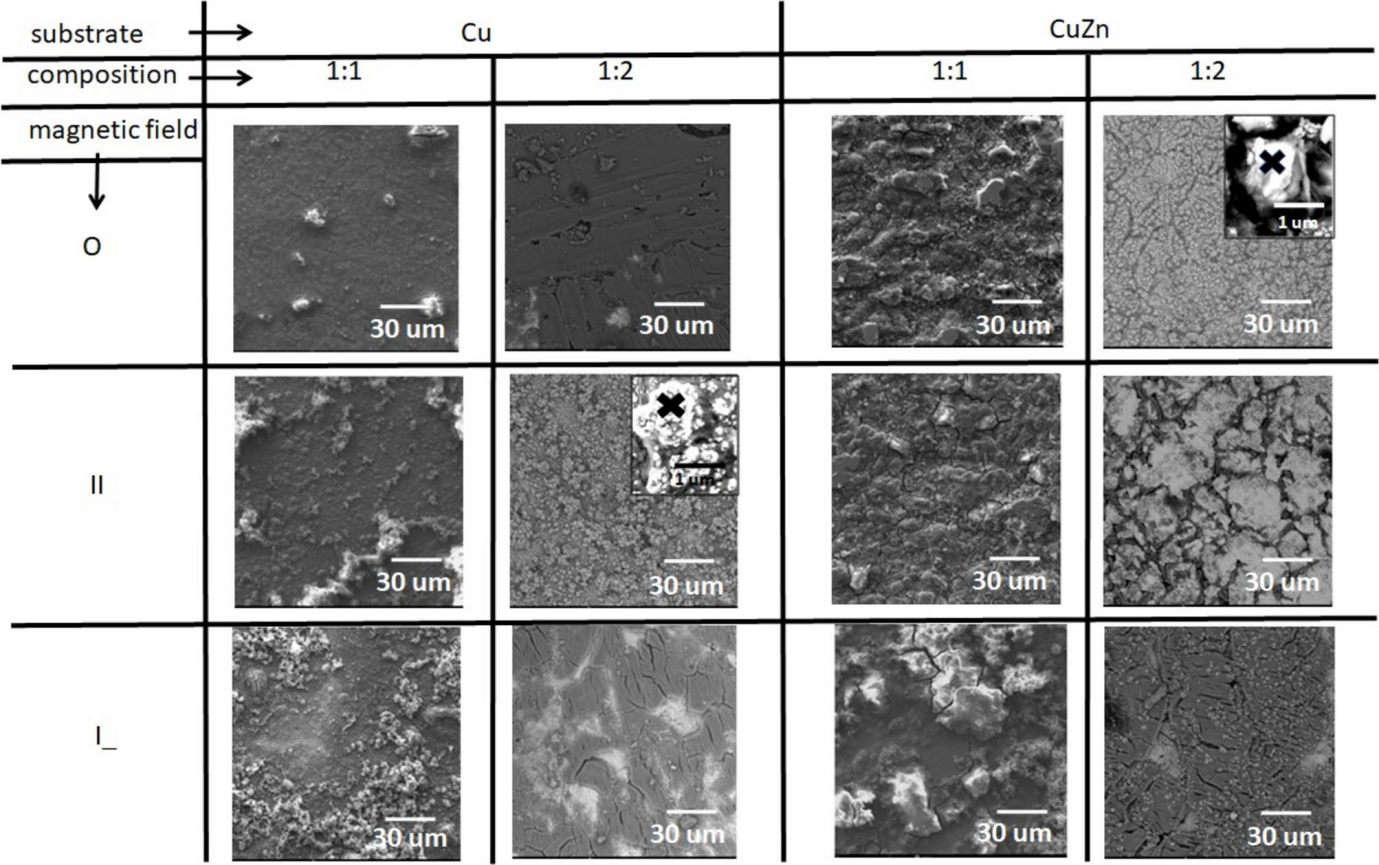
Set of SEM images of the deposited films with and without EMF: time of deposition—3600 s; different substrates (Cu, CuZn) and Fe:Ni ratio; current density—50.0 mA/cm^2^; insets—magnification of islands present on the film.

In Fig. 2, a set of SEM images of fabricated films are depicted with respect of solution composition, used substrate, and external magnetic field orientation.

In the case of films grown on Cu substrate using electrolytes with a 1:1 Fe to Ni ratio, the flat film is covered by larger forms that are situated randomly far away from each other. The clusters are very irregular in shape and vary in size. The presence of an external magnetic field results in the occurrence of a larger number of islands, which are randomly distributed over the whole surface. This can be explained via the magneto-hydrodynamic effect, which increases the ionic mass transfer, and the overgrowth of the number of nucleation centers is observed (Fig. 2). In the case of a 1:2 (Fe to Ni) ratio, resultant films grow differently. Here, only the surface obtained without the presence of EMF is very similar to the previous case. The other two samples show a much larger number of islands that cover the surface more densely and evenly than in the (1:1) series, which results generally in flatter films. Alloys deposited on CuZn substrate do not grow similarly. The resulting films are densely covered by deposits in the form of flat islands on the surface (Fig. 2). Change in the electrolyte composition (1:2) results in modification of coverage, and cracks of films in all three depicted cases are seen (0, II, I_). However, the deposited films are, on average, the flattest of all other cases ^8,31^.

### Composition determination—energy dispersive X-ray spectroscopy

**Fig. 3:**
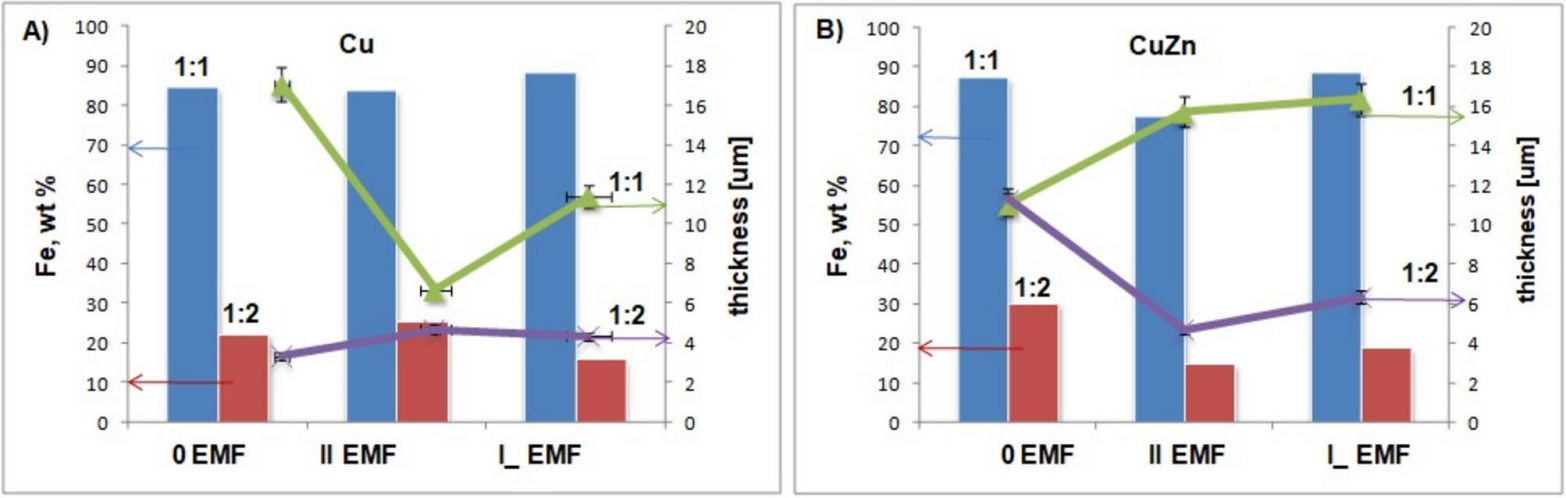
The Fe content and the thickness of the FeNi alloys deposited on two different substrates: current density—50.0 mA/cm^2^; 1:1—left columns (blue), green line, 1:2—right columns (red), violet line; (**A**) Cu, (**B**) CuZn.

Based on elemental analysis performed by EDX, observed changes in composition can be a consequence of substrate, nominal elemental (Fe:Ni) ratio or presence and orientation of the external magnetic field. The collected results are depicted in Fig. 3. Numerical values are presented in Table 1.

**Table 1:** Summary of the research results.

	Cu						CuZn					
Molar ratio	1:1			1:2			1:1			1:2		
EMF orientation	0	II	I_	0	II	I_	0	II	I_	0	II	I_
Fe content [± 1%]	85	84	88	22	26	16	87	78	89	30	15	19
Ni content [± 1%]	15	16	12	78	74	84	13	22	11	70	85	81
Thickness [± 0.5 µm]	17.07	6.69	11.38	3.36	4.70	4.36	11.05	15.73	16.40	11.33	4.70	6.37
Microhardness HV0.1 [± 20]	273	200	211	154	106	148	161	100	271	123	205	153
Depth of wear track [± 0.6 µm]	9.72	4.62	11.15	2.56	2.57	1.94	6.42	12.49	9.45	3.10	2.80	2.44
COF ± 5%	0.82	0.93	0.86	0.65	0.26	0.36	0.44	0.57	0.74	0.54	0.75	0.82
S_ku_ ± 5%	2.91	5.50	6.56	15.60	8.03	31.11	6.64	3.78	5.78	14.70	12.62	15.92
S_sk_ ± 5%	0.33	0.91	1.70	0.45	0.95	0.38	1.67	0.51	1.32	− 0.05	0.03	− 0.33

#### Cu substrate

In the case of the electrolyte with a 1:1 Fe to Ni ratio, typical anomalous deposition (higher content of Fe in the alloy is registered than its presence in the electrolyte). For the second composition 1:2, this effect was not observed. Here, the content of Fe is lower than expected (for details, see Table 1 and Fig. 3). However, the deviation of the composition from nominal is not so significant. When the thickness of the deposited layer is considered, a decreasing trend is visible after the application of EMF (in samples Fe:Ni1:1) and an opposite trend (increase) for deposition in an electrolyte with a composition of 1:2 Fe to Ni.

#### CuZn substrate

The situation regarding the layer composition of the 1:1 Fe to Ni ratio is similar to the one given above. However, the thickness analysis shows an opposite trend change after EMF application for both electrolyte compositions compared to the films obtained on Cu substrate. In 1:1 Fe: Ni film composition the thickness of the deposited layer increases, while in ratio of 1:2 this value in the presence of the EMF decreases^39^.

### Crystallinity determination—X-ray diffraction

**Fig. 4:**
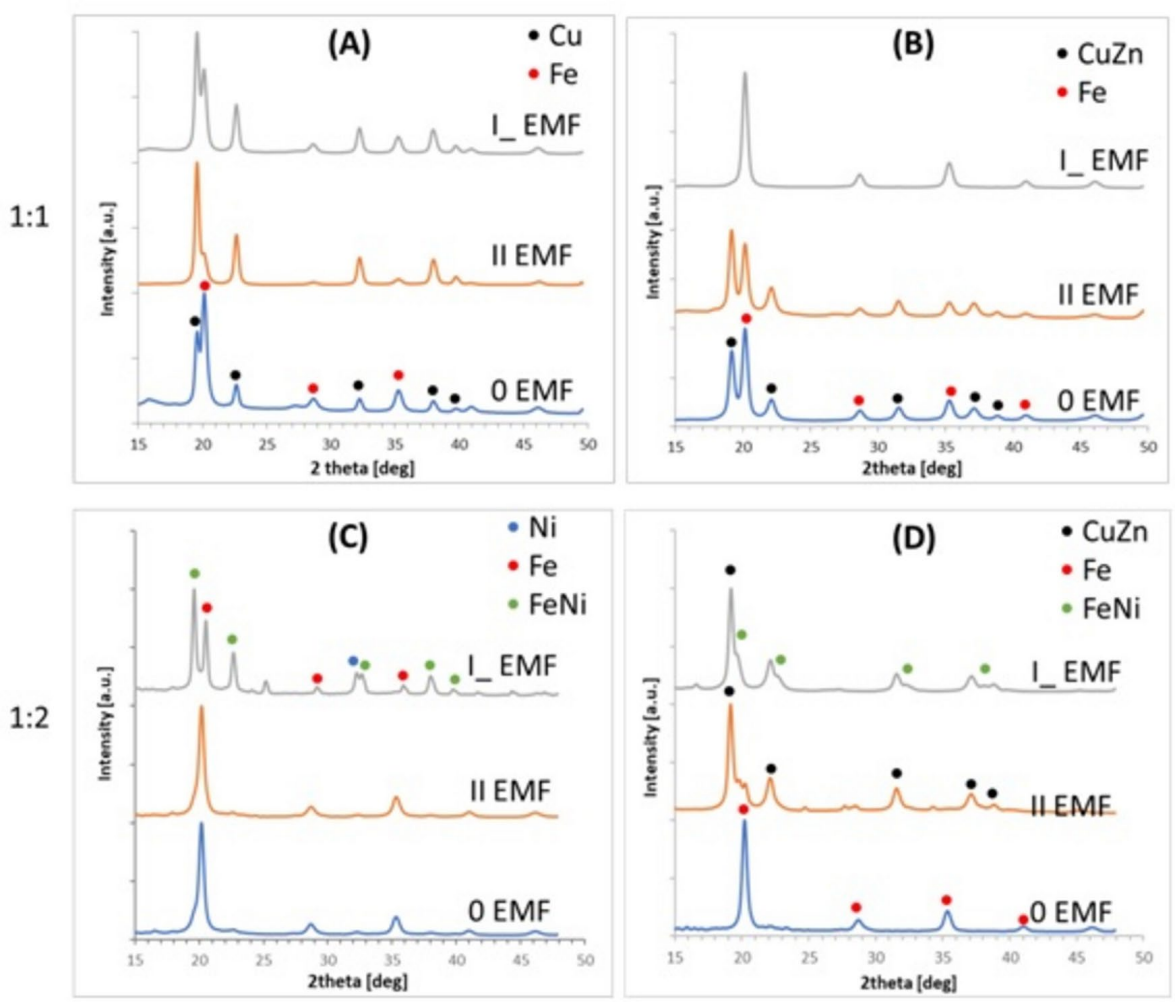
X-ray diffraction patterns of respective samples with respect to the type of substrate and Fe:Ni molar ratio; (**A**, **C**)—Cu substrate; (**B**, **D**)–CuZn substrate.

The results obtained based on the diffraction measurements were gathered and presented in series in Fig. 4.

Presented in Fig. 4, diffraction patterns registered for deposited FeNi layers differ between each other with respect to substrate, solution composition, and EMF presence and orientation. The upper row of FeNi films from the 1:1 solution molar ratio is depicted in the (A) and (B) frame sets, respectively, Cu and CuZn substrates. In all cases presented here, only pure bcc-Fe structures (red dots) with some residual diffraction maxima, which are typical for Cu or CuZn substrates (black dots), can be seen. That analysis correlates well with film composition (Table 1 and Fig. 3). Observed in the diffractograms, maxima can be assigned to bcc-Fe crystal structure with the following Miller indexes: (110) (200) (211) (220), with space group *Im3m*^40^. Accordingly, Miller indexes of Cu or CuZn substrates are recognized as (111) (200) (220) (311) (222) with *Fm3m* space group^41^. The bottom row depicts plots of X-ray diffraction patterns of the FeNi layers from the solution with a 1:2 Fe:Ni molar ratio (respectively Cu frame (C) and CuZn frame (D)). Here, besides bcc-Fe, Cu and CuZnadditional signals are observed, which can be assigned to fcc-FeNi (green dots) or fcc-Ni (blue dot) crystal structures. FeNi alloys are observed only in the case of perpendicular EMF orientation. Here, Miller indexes are (111) (200) (220) (311) (222)s^42^.

### Mechanical analysis

**Fig. 5:**
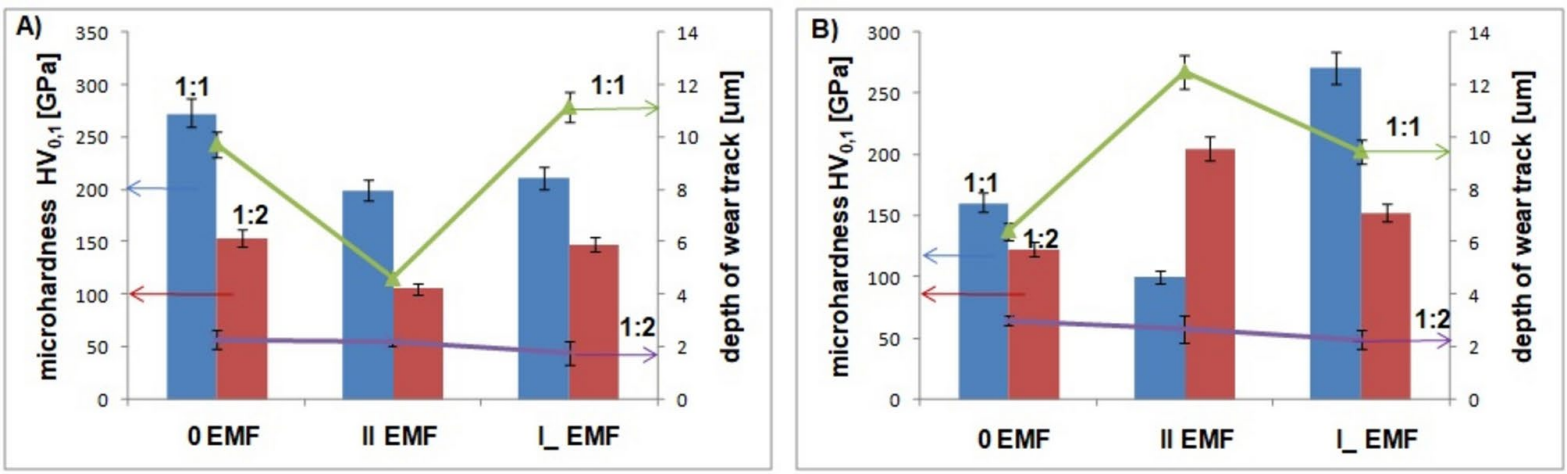
Values of microhardness of tested surfaces and depth of the friction marks for FeNi coatings; 1:1—left columns (blue), green line; 1:2—right columns (red), violet line; (**A**) Cu and (**B**) CuZn substrates.

Figure 5 presents values of microhardness and depth of wear track. This is correlated with film composition. In the case of Fe:Ni ratio 1:1, microhardness is bigger due to higher iron content, which is connected with the anomalous Fe co-deposition. However, for 1:2 Fe:Ni ratio the smaller (normal) alloy’s iron content results in decrease of the layer’s microhardness. This may be due to the increased nickel content in the deposit (and in the electrolyte) which influence the layer’s growth (islands on the film’s surface—Fig. 2).The growths could be easier destroyed and the value of the microhardness is lower.

**Fig. 6:**
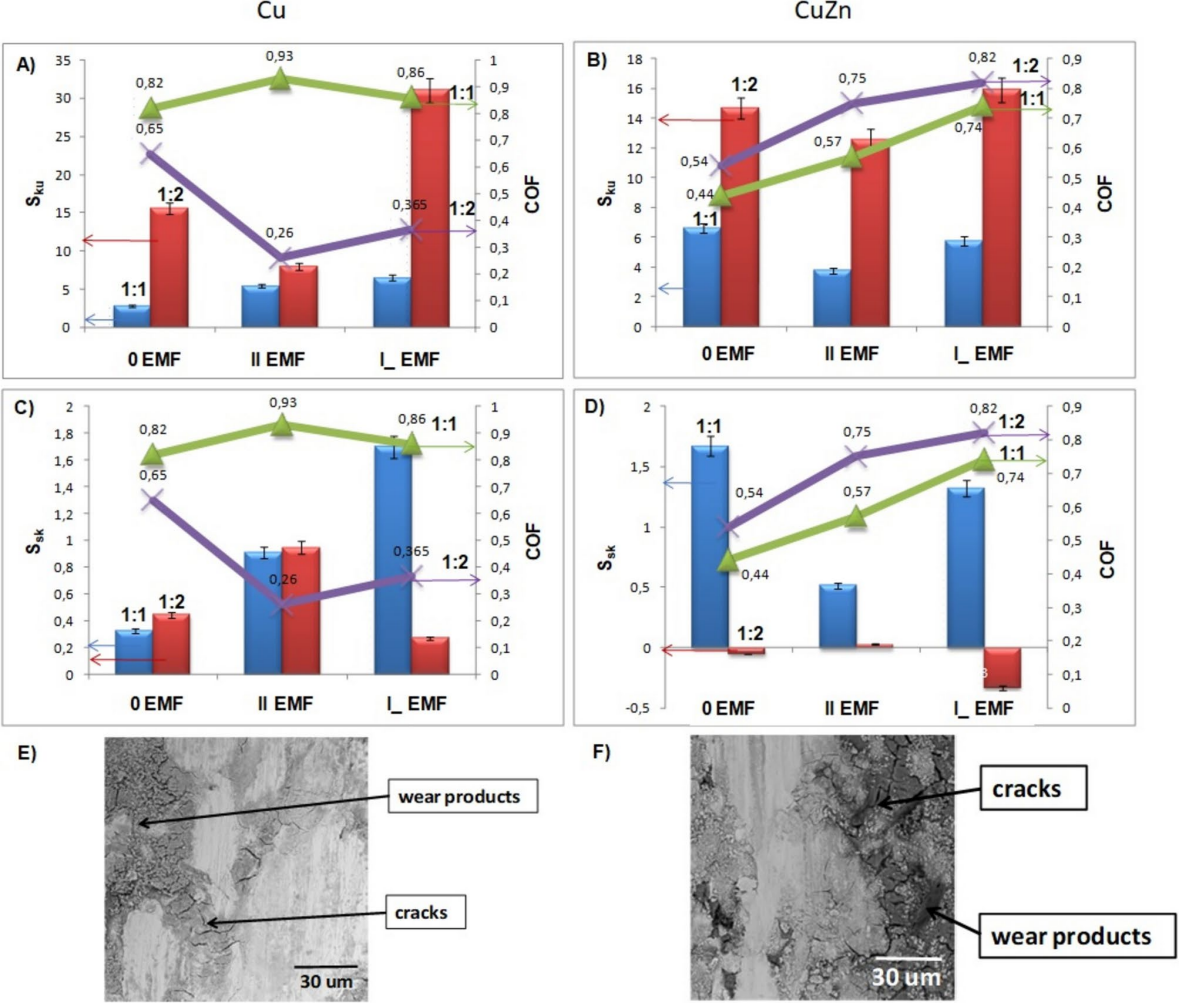
Values of the roughness (S_ku_, S_sk_), wear parameter COF and sections of worn layers of tested surfaces, respectively: 1:1—left columns (blue), green line; 1:2—right columns (red), violet line; (**A**, **C**, **E**) Cu; (**B**, **D**, **F**) CuZn.

Figure 6 presents the roughness and wear parameter COF plot together with respective SEM images of mechanically degraded films.

#### Cu substrate

Higher values of S_ku_ in 1:2 Fe:Ni electrolyte composition than in 1:1 Fe:Ni suggest spiky surfaces and sharpness of the profiles. On the contrary, the value of the COF presents an opposite trend. The friction coefficients for 1:2 Fe:Ni is lower than for 1:1. The film is uniformly covering the surface (based on values of S_sk_), except in the I_ EMF presence.

#### CuZn substrate

Films obtained for 1:2 Fe:Ni are more pointed than in the case 1:1. Values of the COF for 1:2 Fe:Ni are slightly higher than for 1:1. In the case of 1:2 Fe:Ni composition, surfaces with more valleys than peaks are obtained.

The relationship between these quantities can be discussed based on EDX analysis (inset on Fig. 2) and film thickness measurements (Fig. 3). A point elemental analysis allows the determination of the alloy composition in selected fragments on the sample surface (island growth). The spot marked as X (Fig. 2—insets) showed a higher content of Ni in the alloy, which accelerated the growth rates of deposits^39^. This, in turn, resulted in significant changes in the thickness of the deposited layer. The kurtosis values give feedback on the planarity of the obtained surfaces. The smoother the surface, the lower the friction coefficient. It is especially visible in the case of II EMF on the Cu substrate (1:2)—Fig. 6. Literature studies have shown that the boric acid presence improved the lateral and outward growth during the deposition^43^. Presented in this paper cases are examples of the decrease in the microhardness values (Fig. 5). It is highly correlated with the Ni reach coating in the cases of 1:2 electrolyte composition (II EMF on Cu substrate and 0 EMF on CuZn substrate). It was reported that the coating’s composition results in changes in the microhardness value^39^.

The effect of cracks (Fig. 2) on the alloy surface (0 EMF, II EMF, I_ EMF)can be found in the measured values of the S_sk_ and S_ku_ (measures of asymmetry), especially on the CuZn substrate (Fig. 6)^8,31^.

Some authors discussed a directly proportional relationship between the alloy’s nickel content and the friction coefficient’s value^39^. Presented here, analysis of the composition of all alloys in connection with the COF parameter showed such a relationship only in the case of a 1:2 Fe:Ni ratio in the electrolyte.

**Fig. 7:**
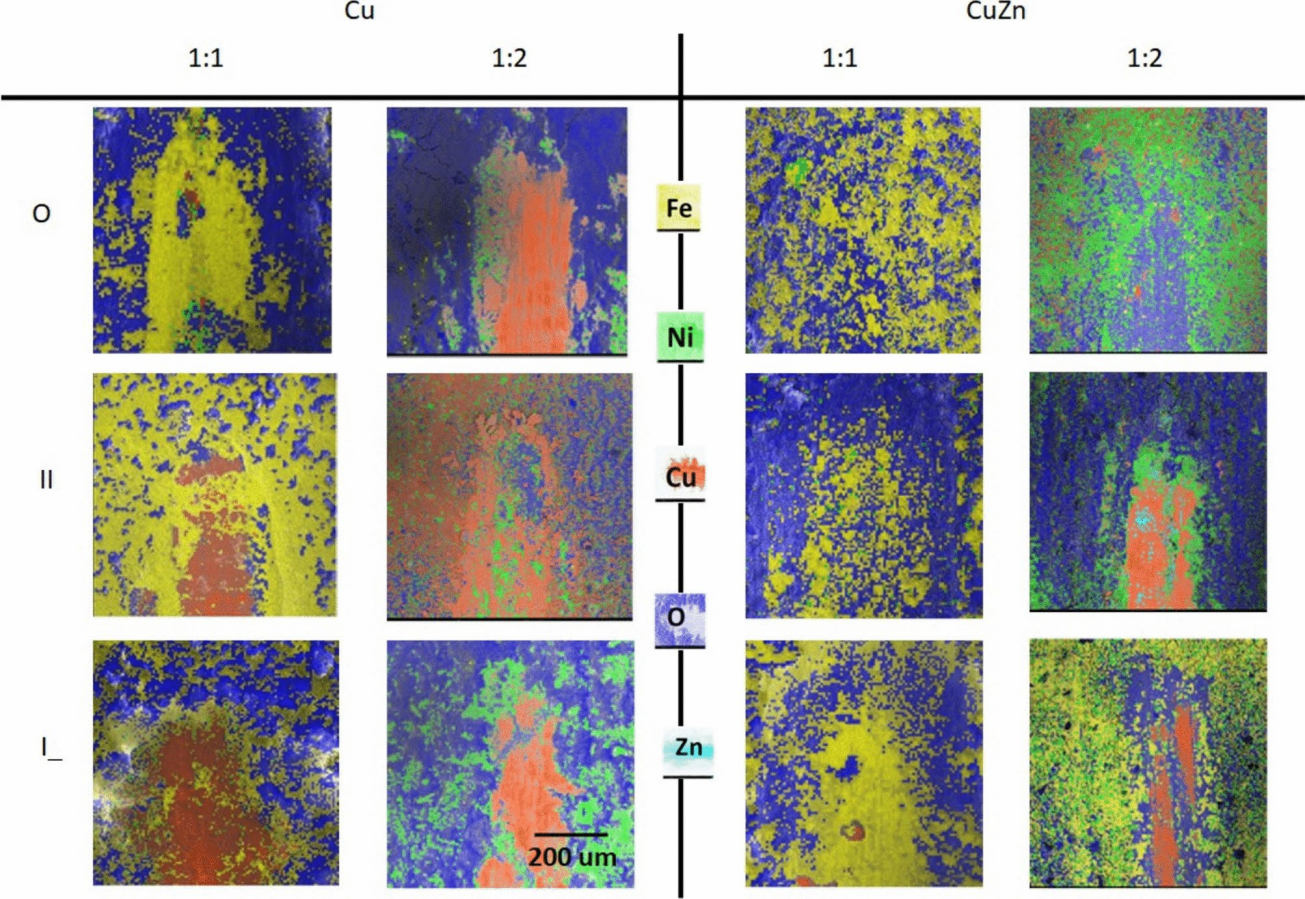
SEM–EDX analysis of wear tracks for Cu and CuZn substrates; Current density—50.0 mA/cm^2^; deposition time—3600 s.

Figure 7 presents the analysis of friction traces for all tested cases. The situation is very similar for both substrates (Cu, CuZn). The thinner the layer, the more significant tracks on the surfaces can be seen.

#### Cu substrate

The thinner FeNi layer (4–6 μm) results in significant substrate exposure after friction. That is especially clear in the case of 1:2 Fe:Ni electrolyte composition. The other sample (1:1 Fe:Ni), due to the presence of thicker film (12–16 μm), is not showing so clear tracks.

#### CuZn substrate

A similar situation was revealed of friction traces in both electrolytes. The images of the traces in the case of layers deposited in the 1:2 Fe:Ni electrolyte show the substrate elemental composition. For the thicker layer (12–16 μm) obtained for the 1:1 Fe:Ni ratio, the layer breaking down to the substrate is not registered.

The spiky surface (higher S_ku_ value—Fig. 6) is more easily abraded, resulting in more effective uncovering of the substrate. This effect was observed for both substrates in the case of 1:2 Fe:Ni ratio—Fig. 7.

In order to provide a broader perspective on all the research results, table with numerical values of Fe and Ni content, layer thickness, microhardness, depth of wear, S_km_, S_sk_ and COF are presented (Table 1).

## Conclusions

Receiving the final product is always preceded by a multi-stage production process. However, in many cases, the condition of the product’s surface needs to be addressed since its roughness affects many properties. These, in turn, result in using (or not) a given element in industrial production. The analysis of the data in the article showed that the electrolyte composition significantly impacts the obtained FeNi layer. The authors demonstrated remarkable differences in the morphology, composition, and tribological properties of obtained films. The layers obtained from the 1:1 Fe:Ni electrolyte composition (compared to 1:2 Fe:Ni) contain an increased amount of Fe in content up to 88%, in comparison to nominal—33% (anomalous deposition) and therefore the presence of a Fe crystal structure (based on X-ray diffraction). As a result, layers are characterized by increased microhardness of 200–273 μm (compared to 106–154 μm) and thickness of 6.69–17.07 μm (compared to 3.36–4.70 μm). This is also reflected in a lower value of the S_ku_ 2.91–6.56 (compared to 8.03–31.11) as well as corresponding to this value of the friction coefficient 0.82–0.93 (compared to 0.26–0.65)—worse abrasion. Additionally, the use of an external magnetic field (particularly one of its settings) revealed an outstanding impact on the analyzed alloy parameters –composition, COF, depth of wear tracks or thickness. This generated additional convection and a reduction in the thickness of the diffusion boundary layer. The analysis of the experimental results clearly showed that the (1:1) and the (1:2) electrolyte composition show different trends of changes in the film composition as well as in the technical parameters mentioned above values.

## Data Availability

The datasets used and/or analysed during the current study available from the corresponding author on reasonable request. The data that support the findings of this study are available from the Centre for Synthesis and Analysis of BioNanoTechno at the University of Białystok which was co-financed by the European Union under the Operational Programme Development of Eastern Poland (POW.01.03.00-20-034/09-00), but restrictions apply to the availability of these data, which were used under license for the current study, and so are not publicly available. Data are however available from the authors upon reasonable request and with permission of the Centre for Synthesis and Analysis of BioNanoTechno at the University of Białystok.
